# Lipoprotein subclass profiles in young adults born preterm at very low birth weight

**DOI:** 10.1186/1476-511X-12-57

**Published:** 2013-04-30

**Authors:** Petteri Hovi, Eero Kajantie, Pasi Soininen, Antti J Kangas, Anna-Liisa Järvenpää, Sture Andersson, Johan G Eriksson, Mika Ala-Korpela, Karoliina Wehkalampi

**Affiliations:** 1 Department of Chronic Disease Prevention, National Institute for Health and Welfare, Mannerheimintie 166, P.O. Box 30, FI-00271 Helsinki, Finland; 2 Children’s Hospital Helsinki University Central Hospital and University of Helsinki, Stenbäckinkatu 11, FI-00029 HUS Helsinki, Finland; 3 NMR Metabolomics Laboratory, School of Pharmacy University of Eastern Finland, P.O. Box 1627, FI-70211 Kuopio, Finland; 4 Unit of General Practice Helsinki University Central Hospital and University of Helsinki, P.O. Box 20, FI-00029 HUS Helsinki, Finland; 5 Folkhälsan Research Center University of Helsinki, Haartmaninkatu 8, Helsinki, FI-00014, Finland; 6 Department of General Practice and Primary Health Care, University of Helsinki, Haartmaninkatu 8, FI-00014 Helsinki, Finland; 7 Computational Medicine, Institute of Health Sciences, University of Oulu and Oulu University Hospital, Aapistie 5 A, P.O.Box 5000, 90014 Oulu, Finland; 8 Computational Medicine, School of Social and Community Medicine University of Bristol, Oakfield House, Oakfield Grove, Bristol BS8 2BN, UK

**Keywords:** Very low birth weight, Prematurity, Lipoprotein, Lipids, Subclass

## Abstract

**Background:**

Adults born preterm at very low birth weight (VLBW ≤ 1500g) have increased risk factors for cardiovascular diseases including high blood pressure and impaired glucose regulation. Non-optimal lipoprotein profile is generally also likely to affect the increased cardiovascular risk, but lipoprotein subclass level data on adults born at VLBW are sparse.

**Subjects and methods:**

We studied 162 subjects born at VLBW and 169 term-born controls, aged 19 to 27 years. Total lipid, triglyceride and cholesterol concentrations of 14 lipoprotein subclasses were determined by proton nuclear magnetic resonance spectroscopy in the fasting state and in 2-hour serum samples from an oral glucose tolerance test.

**Findings:**

In comparison to controls, VLBW subjects had significantly higher fasting concentration of triglycerides in chylomicrons and largest very-low-density lipoprotein particles [XXL-VLDL-TG, difference 0.026 (95% CI: 0.004 to 0.049), P = 0.024], and of triglycerides in small high-density lipoprotein particles [S-HDL-TG, 0.026 (95% CI: 0.002 to 0.051), P = 0.037]. The seemingly important role of triglycerides was further supported by principal component analysis in which the first component was characterized by multiple lipoprotein triglyceride measures.

**Conclusions:**

Young adults born at VLBW and their peers born at term had triglyceride-related differences in both VLDL and HDL subclasses. These differences suggest that the increased risk factors for cardiovascular diseases among the VLBW individuals in adulthood may partly relate to impaired triglyceride metabolism.

## Background

Adult-onset cardiovascular diseases have their origins partly in intrauterine and early postnatal life [1]. As young adults, very low birth weight (VLBW, ≤1500 g) infants have increased risk factors for cardiovascular diseases and type 2 diabetes [2-6]. They have, e.g., higher blood pressure than their term-born peers, and higher indexes of insulin resistance and glucose intolerance [2-6]. Some, although not all, studies have shown associations between preterm birth and a higher fat percentage in adulthood [5,7]. Findings on serum lipid profile are inconsistent. Although some studies have indicated that low gestational age at birth relates to non-optimal serum lipids in later life [8-10], most have not reported dyslipidemia in adults born preterm [2,5,7,11-13]. However, these studies have been limited to concentrations of lipids conventionally measured in clinical practice - total, low-density lipoprotein (LDL) and high-density lipoprotein (HDL) cholesterol and total triglycerides, whereas lipoprotein subclasses may provide a more precise indicator for atherogenicity [14,15].

Here we investigated lipoprotein metabolism at subclass level in VLBW and control subjects from the Helsinki Study of Very Low Birth Weight Adults by proton nuclear magnetic resonance (NMR) spectroscopy.

## Subjects and methods

The participants are from a longitudinal follow-up cohort of subjects born preterm at VLBW between 1978 and 1985 and discharged alive form the neonatal intensive care unit of Children's Hospital, Helsinki University Central Hospital, Finland. Of the original 335 survivors, 255 VLBW subjects lived in the greater Helsinki area at the time of follow-up and were invited to a clinical study together with a sex-, age-, and birth hospital-matched comparison group of 314 term-born subjects who were not small for gestational age (birth weight more than −2 SD) [16]; 166 VLBW and 172 controls participated. Of these, 4 and 3, respectively, were excluded because of not having fasted overnight, or being pregnant. Thus, the study finally included 162 VLBW and 169 term-born control subjects. None of these were treated with lipid lowering medication. One subject had type 1 diabetes. All subjects underwent a 75 g 2-hour oral glucose tolerance test (OGTT), based on which none had type 2 diabetes [5].

After an overnight fast of at least 8 hours, the clinical examination included weight and height measurement, and a 2-hour 75 g OGTT [5]. From both baseline- and 2-hour-sample, blood was drawn for lipoprotein subclass analysis. Moreover, the participants completed questionnaires that covered their medical history, smoking habits, and educational level of their parents.

Total lipid (L), triglyceride (TG) and cholesterol (C) concentrations of 14 lipoprotein subclasses were analyzed by proton NMR spectroscopy in serum samples. The details of this methodology have been described [17,18] and this platform has recently been applied in large-scale epidemiological and genetic studies [19,20]. The lipoprotein subclass data are as follows: chylomicrons and largest (XXL) very-low-density lipoprotein (VLDL) particles (average particle diameter at least 75 nm); five different VLDL subclasses: very large (XL) VLDL (average particle diameter of 64.0 nm), large (L) VLDL (53.6 nm), medium (M) VLDL (44.5 nm), small (S) VLDL (36.8 nm), and very small (XS) VLDL (31.3 nm); intermediate-density lipoprotein (IDL) (28.6 nm); three LDL subclasses: L-LDL (25.5 nm), M-LDL (23.0 nm), and S-LDL (18.7 nm); and four HDL subclasses: XL-HDL (14.3 nm), L-HDL (12.1 nm), M-HDL (10.9 nm), and S-HDL (8.7 nm). Due to resolution and concentration issues TG and C are not available for every subclass [20]. The mean size for VLDL, LDL, and HDL particles was calculated by weighting the corresponding subclass diameters with their particle concentrations. IDL particles were included in the LDL measure. Altogether 44 lipoprotein measures were used.

Statistical analyses were conducted using SPSS 17 and 19 software (IBM SPSS, Chicago, IL). Comparison of basic characteristics between VLBW and term was performed using *t*-test for continuous and chi-square -test for categorical variables. Because of skewed distributions lipoprotein subclass measurements were transformed as follows: 0.1 was added to avoid zero-values and natural logarithm was used to achieve more symmetrical distributions. Distributions were checked after logarithm transformation and those two that were not sufficiently normal were transformed again. To find a situation where the residuals of XXL-VLDL-TG and residuals of XXL-VLDL-L were fairly normal, we utilized the ‘Box-Cox transformations for linear models’ in the ‘car’-package downloaded in April 4^th^, 2013, to R-software, version 2.15.2. Oct 26, 2012. To avoid non-positive dependent values we added 0.1 to the dependent prior to the Box-Cox iterative procedure. The *lambdas* with maximal log likelihood were -7.3 and -5.3, for the respective outcomes, yielding to our best transformations for the full models: y’ = (y + 0.1)^(*lambda*-1)^/(*lambda*), where y = original variable. With these y’ as dependent in the full models, the resulting in symmetric distributions of residuals but, unfortunately, with less pronounced aggregation of mid values.

We used multiple linear regression to compare individual lipoprotein subclass measurements between VLBW and control subjects. We adjusted for confounding factors in different models. The fully adjusted model included age, sex, height, highest parental education, body mass index, mother’s smoking during pregnancy, and daily smoking of the participant (yes/no) as covariates. Additionally we also adjusted for alcohol use in the fully adjusted model; dicotomized values for whether the subject used alcohol weekly to get drunk or not, and whether the subject ever used alcohol or not. We also adjusted for fasting and 2-hour glucose concentrations in additional models. We tested group differences in 44 outcome variables. Many of the lipoprotein measures are highly correlated and thus we also utilized the *principal component analysis* to reduce the number of outcomes tested (the FACTOR-program in SPSS, version 19, was used). We included the five first components as they explained 95% of the variation and only their eigenvalues were greater than 1.0. We then tested group differences (VLBW versus term) of each individual's Varimax-rotated component scores using adjusted linear regression models.

The study was performed according to the declaration of Helsinki. The study protocol was approved by the Ethics Committee at the Helsinki and Uusimaa Hospital District. Written informed consent was obtained from each participant.

## Results

**Figure 1:**
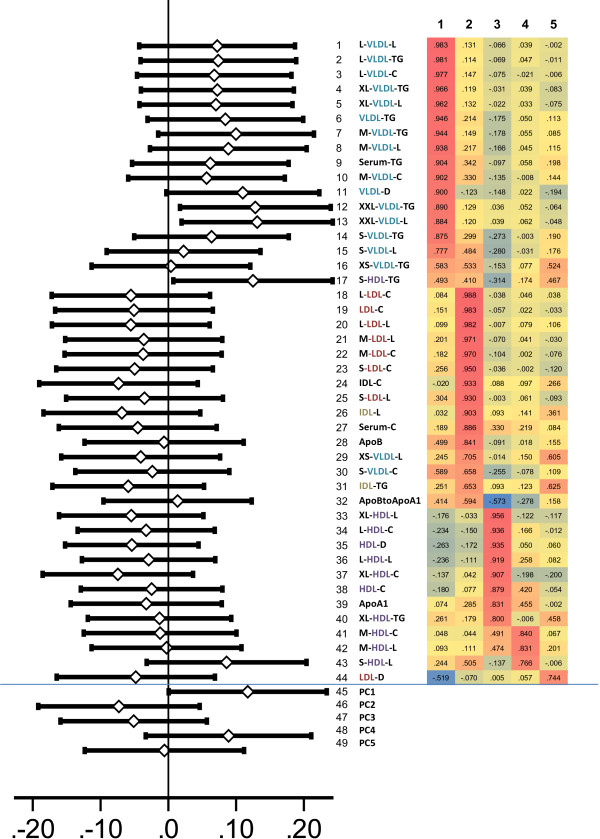
**Middle: All the 44 lipoprotein subclass measures analysed.** Left: A forrestplot of fully adjusted differences in fasting serum samples between VLBW and term subjects. Right: Rotated Component Matrix; using principal component analysis all lipoprotein measures were re-organized into 5 components. Bottom: Factor scores from 5 components that were entered into multiple linear regression models. Vertical line at zero indicates no difference, larger values for VLBW subjects are positive differences. (VLBW, very low birth weight, ≤1500 g; HDL, high-density lipoprotein; LDL, low-density lipoprotein; IDL, intermediate-density lipoprotein; VLDL, very-low-density lipoprotein; TG, triglycerides; C, total cholesterol; L, total lipid; PC, principal component).

VLBW subjects were (Table 1). Using the unadjusted model, there were no differences in the separate 44 fasting lipoprotein measures between VLBW and term (data not shown). Fully adjusted regression models for each measure (Figure 1) showed no group differences in most values. However, compared with controls, VLBW subjects had significantly higher concentration of triglycerides in chylomicrons and largest VLDL particles [XXL-VLDL-TG, difference 0.026 (95% CI: 0.004 to 0.049), P = 0.024] and higher total lipids in these particles [XXL-VLDL-L, difference 0.036 (95% CI 0.005 to 0.037), P = 0.021]. They also had higher triglycerides in small HDL particles [S-HDL-TG, difference 0.026 (95% CI: 0.002 to 0.051), P = 0.037]. No differences in these parameters were observed in the 2-hour OGTT samples (data not shown).

**Table 1:** Characteristics of the study participants

**Characteristic**	**VLBW ( *n * = 161)**	**Term (*n *= 163)**	***P***
Women, n (%)	92 (57)	97 (60)	0.8
Men, n (%)	69 (43)	66 (41)	0.8
At birth			
Gestational age, mean (SD), wk	29.1 (2.2)	40.1 (1.2)	<0.0001
Birth weight, mean (SD), g	1117 (220)	3575 (471)	<0.0001
Birth weight SDS, mean (SD), SDS	−1.3 (1.5)	0.0 (1.0)	<0.0001
SGA, n (%)	54 (34)	0	
Parental			
Mother’s smoking during pregnancy	30 (19)	25 (15)	0.5
Parental education, n (%)			0.5
Elementary	17 (11)	11 (7)	
High school	32 (20)	29 (18)	
Intermediate	65 (40)	53 (33)	
University	44 (27)	69 (42)	
At clinical examination			
Age, mean (SD), y	22.4 (2.2)	22.5 (2.2)	0.9
Height, mean (SD), cm			
*Women*	162.1 (7.7)	167.2 (6.8)	<0.0001
*Men*	174.6 (7.8)	180.4 (6.5)	<0.0001
Body mass index, mean (SD), kg/m^2^			
*Women*	22.2 (4.0)	22.8 (3.7)	0.3
*Men*	22.1 (3.7)	23.2 (3.2)	0.06
Daily smoking, n (%)	35 (22)	54 (33)	0.08

VLBW, very low birth weight (≤1500 g).

SGA, small for gestational age (birth weight < −2SD).

XXL-VLDL-TG and XXL-VLDL-L were somewhat skewed to the right still after the log transformation. Thus, we transformed them with BoxCox formulas and analyzed again the effect of VLBW. In result, the stardardized betas for these two variables changed from log transformed values to boxcox values: XXL-VLDL-TG from 0.13 (p = 0.021) to 0.07 (p = 0.236) and XXL-VLDL-L changed from 0.13 (p = 0.019) to 0.13 (p = 0.20).

Adding alcohol as a covariate in the fully adjusted model did not change the observed difference in XXL-VLDL-TG, XXL-VLDL-L, or S-HDL-TG between VLBW subjects and controls. Neither were these differences changed after inclusion of fasting or 2-hour glucose as a covariate in linear regression (Table 2).

**Table 2:** B and 95% confidence interval (CI) and P for the difference in triglycerides in chylomicrons and extremely large VLDL (XXL-VLDL-TG), in total lipids in these particles (XXL-VLDL-L) as well as in small HDL particles (S-HDL-TG) when adjusted for; 1) full model, 2) + alcohol use, 3) + glucose indices

	**XXL-VLDL-TG**		**XXL-VLDL-L**		**S-HDL-TG**	
	**B (95% CI)**	**P**	**B (95% CI)**	**P**	**B (95% CI)**	**P**
1	0.026 (0.004, 0.049)	0.024	0.036 (0.005, 0.037)	0.021	0.026 (0.002, 0.051)	0.03
2a	0.030 (0.007, 0.053)	0.001	0.041 (0.011, 0.072)	0.009	0.030 (0.006, 0.057)	0.016
2b	0.028 (0.005, 0.051)	0.016	0.038 (0.008, 0.069)	0.014	0.026 (0.002, 0.051)	0.036
3a	0.024 (0.001, 0.047)	0.040	0.033 (0.002, 0.064)	0.037	0.024 (−0.001, 0.049)	0.059
3b	0.022 (0.001, 0.044)	0.044	0.031 (0.002, 0.061)	0.040	0.025 (0.001, 0.049)	0.045

1) full model; adjustment for age, sex, height, highest parental education, body mass index, mother’s smoking during pregnancy, and daily smoking of the participant.

2a) full model + adjustment for whether the subject ever drinks alcohol (yes or no).

2b) full model + adjustment for whether the subject uses alcohol to get drunk on weekly basis (yes or no).

3a) full model + adjustment for fasting glucose.

3b) full model + adjustment for 2-hour glucose in oral glucose tolerance test.

We then performed a *principal component analysis*, which re-organized all lipoprotein variables into five uncorrelated components. The rotated component loadings for the lipoprotein variables (tabled in Figure 1) characterize *principal component* 1 (PC1) mainly as a marker of VLDL and triglycerides and PC2 as a marker of LDL, cholesterol and apolipoprotein B (ApoB). In a linear regression model, scores for PC1 were higher in VLBW subjects than in control subjects [standardized difference 0.12 (95% CI: 0.00 to 0.23), P = 0.049] (Figure 1). *Principal component analysis* for 2-hour OGTT samples yielded similarly to five components. Regarding these components, VLBW and control subjects scored similarly (P-values ≥ 0.47).

## Discussion

We observed that, in comparison to controls, adults born at VLBW have higher concentrations of triglycerides both in the largest VLDL particles and in small HDL particles. These potentially atherogenic characteristics of the lipid profile [21] may contribute to an increased risk of cardiovascular disease in adults born preterm at VLBW.

In the general population a weak negative correlation has been reported between birth weight and serum total cholesterol [22]. Similarly, an inverse correlation has been reported between gestational age and serum triglycerides in 11-15-year-olds [8], total cholesterol in 44-45-year-old women [9], and LDL cholesterol and ApoB among 16-year-old boys [10]. Based on previous studies on preterm-born subjects, conventionally measured lipid levels in fasting blood samples are, however, similar as in term-born individuals [2,5,7,11-13]. For instance, in the current cohort the levels of total, and HDL cholesterol, as well as serum triglycerides do not differ between the VLBW subjects and controls [5]. However, triglycerides in the largest VLDL and small HDL particles are higher in the VLBW than in the term-born participants. These new extensive lipoprotein data thus suggest particular impairments in triglyceride metabolism in relation to VLBW. As triglyceride-mediated pathways are causally related to coronary heart disease [23] these findings may partly explain the increased risk for cardiovascular diseases among the VLBW individuals in adulthood and also lend support for further studies with more detailed lipoprotein measures [14,15].

A strength of this study is the measurement of extensive lipoprotein subclass data at both in the fasting state and 2-hours after an oral glucose challenge. We were also able to adjust for most potential confounding factors, such as body mass index, smoking, and parental education. These factors did not explain the observed difference between VLBW and control subjects. A common challenge, as also here, in the studies of VLBW individuals is the relatively small number of study participants available. Combined with the rather high number of lipoprotein measures analysed, this created a statistically difficult situation; however, the trends for biologically related variables supported the findings as well as reduction of the outcome variables using principal component analysis. The remaining skewedness in the log-transformed variables may, in general, cause false negative findings. During the primary analysis, we further ‘Box-Cox transformed’ the variables when it was suspected logarithmic transformation did not adequately remove the skewedness. After this further transformation the difference estimates did not increase, but decreased, approximately by 50% at maximum. Thus, these two methods, together with the principal component approach, support VLBW adults exhibit a difference in the extremely large VLBW triglycerides and total lipids.

In conclusion, we found atherogenic characteristics in the lipoprotein profile of young adults born preterm at VLBW. The differences between the VLBW and the term-born individuals were not detectable with conventional lipid measurements. These findings, though subtle, may indicate that impaired triglyceride metabolism partly contributes to the increased risk for cardiovascular disease among adults born severely preterm.

## Abbreviations

ApoA1: Apolipoprotein A-I; ApoB: Apolipoprotein B; C: Total cholesterol; D: Diameter; HDL: High-density lipoprotein; IDL: Intermediate-density lipoprotein; LDL: Low-density lipoprotein; L: Total lipids; NMR: Nuclear magnetic resonance; OGTT: Oral glucose tolerance test; PC: Principal component; SGA: Small for gestational age; TG: Triglycerides; VLBW: Very low birth weight; VLDL: Very-low-density lipoprotein.

## Competing interests

The authors declare that they have no competing interests.

## Authors’ contributions

KW took part in carrying out the statistical analyses, participated in planning of the study, and drafted the manuscript. PH took part in carrying out the statistical analyses, and participated in writing the manuscript. PH, EK, SA, A-LJ, and JGE participated in the design of the clinical study. PH, EK, SA, and A-LJ collected the study participants and PH, EK, SA, and JGE performed the clinical studies. PS, AK, and MA-K designed and performed the NMR analyses. MA-K participated in the statistical interpretations as well as in writing the manuscript. All authors read and approved the final manuscript.
